# Supplementary material for: Osteoblast/osteocyte-derived interleukin-11 regulates osteogenesis and systemic adipogenesis
Source: Nat Commun. 2022 Nov 23;13:7194. doi: 10.1038/s41467-022-34869-3 (PMC9691688; doi:10.1038/s41467-022-34869-3)
Supplement: Supplementary file 1 — Supplementary Information [file 41467_2022_34869_MOESM1_ESM.pdf]

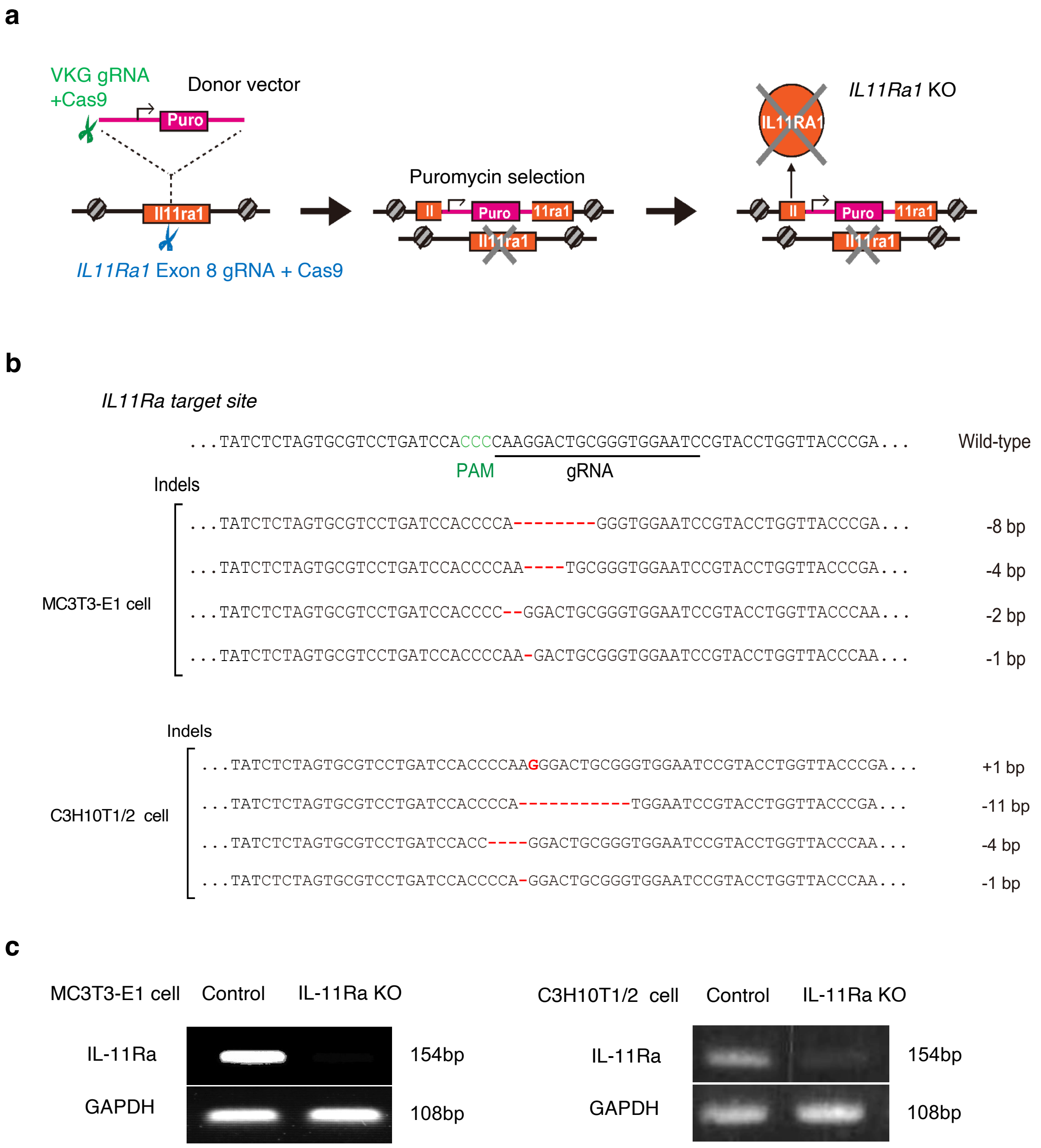

Supplementary Figure 1. Schematic representation of CRISPR/Cas9-mediated knock-out using versatile NHEJ-based knock-in modules for genome editing (VIKING).
(a) The experimental workflow of the VIKING method. The system comprises three components: a donor vector, having an antibiotic-resistance gene as a selection marker (*Puro*, magenta); a donor cleaving vector (green); and a locus-specific cleaving vector that cleaves an exon of the *interleukin 11 receptor alpha chain 1* (*IL-11ra1, Exon 8*) (blue). The *IL-11ra1* exon is destroyed by the insertion of donor vector and the indels caused for cleavage by gRNA-Cas9 complexes. **(b)** Conformation of deletion variants of the *IL-11ra* gene in the knocked-out MC3T3-E1 or C3H101/2 cell line. Representative genome sequences of the *IL-11ra1* locus in the knock-out cell lines. Protospacer adjacent motif (PAM) sequence is shown with green. The indels are colored with red. **(c)** IL-11RA expression detected by RT-PCR in IL-11RA knockout and control MC3T3-E1 and C3H10T1/2 cells. Representative figures of three independent experiments.

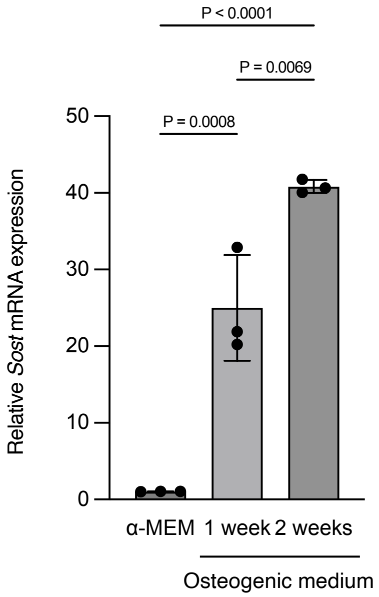

Supplementary Figure 2. Time-dependent increase in sclerostin expression in MC3T3-E1 cells cultured in an osteogenic medium.

Expression levels of *Sost* in MC3T3-E1 cells cultured in an osteogenic medium containing α-MEM supplemented with 50 µg/ml ascorbic acid and 10 mM β-glycerophosphate compared with those in cells cultured in α-MEM alone. n=3. Data are means ± SD. P values are calculated using ordinary one-way ANOVA with Tuckey's multiple comparisons test.

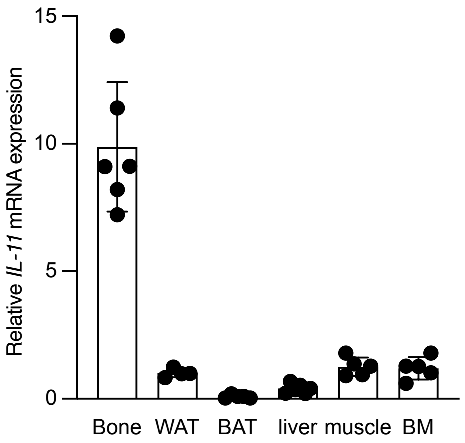

Supplementary Figure 3. IL-11 is expressed mostly in the bone.
Relative expression levels of *IL-11* in the bone (n = 6); WAT, white adipose tissue (n = 4); BAT, brown adipose tissue (n = 5); liver (n = 6); muscle (n = 5); and BM, bone marrow (n = 5). Data are means ± SD.

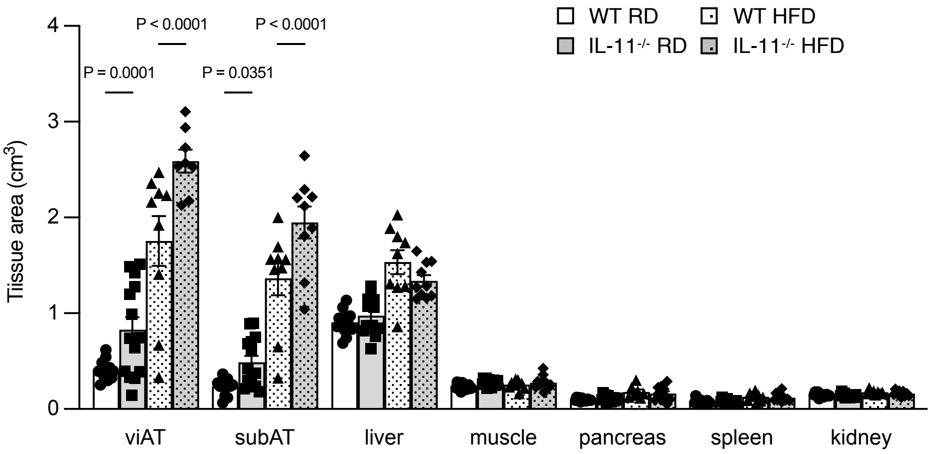

Supplementary Figure 4. Adipose tissue weight of different tissues in WT and IL-11$^{-/-}$ mice under RD and HFD at 24 weeks old.

viAT, visceral adipose tissue of WT on RD (n = 11) or HFD (n = 9) and IL-11^{-}/- mice on RD (n = 14) or HFD (n = 8); subAT, subcutaneous adipose tissue of WT on RD (n = 13) or HFD (n = 9) and IL-11^{-}/- mice on RD (n = 14) or HFD (n = 9); liver of WT on RD (n = 13) or HFD (n = 9) and IL-11^{-}/- mice on RD (n = 14) or HFD (n = 10); pancreas of WT on RD (n = 13) or HFD (n = 9) and IL-11^{-}/- mice on RD (n = 15) or HFD (n = 10): spleen of WT on RD (n = 13) or HFD (n = 7) and IL-11^{-}/- mice on RD (n = 14) or HFD (n = 10); and kidney of WT on RD (n = 13) or HFD (n = 8) and IL-11^{-}/- mice on RD (n = 14) or HFD (n = 9). Data are means ± SD. P values between WT and IL-11$^{-/-}$ mice under the same diet are calculated by two-way ANOVA with Tukey’s multiple comparisons test.

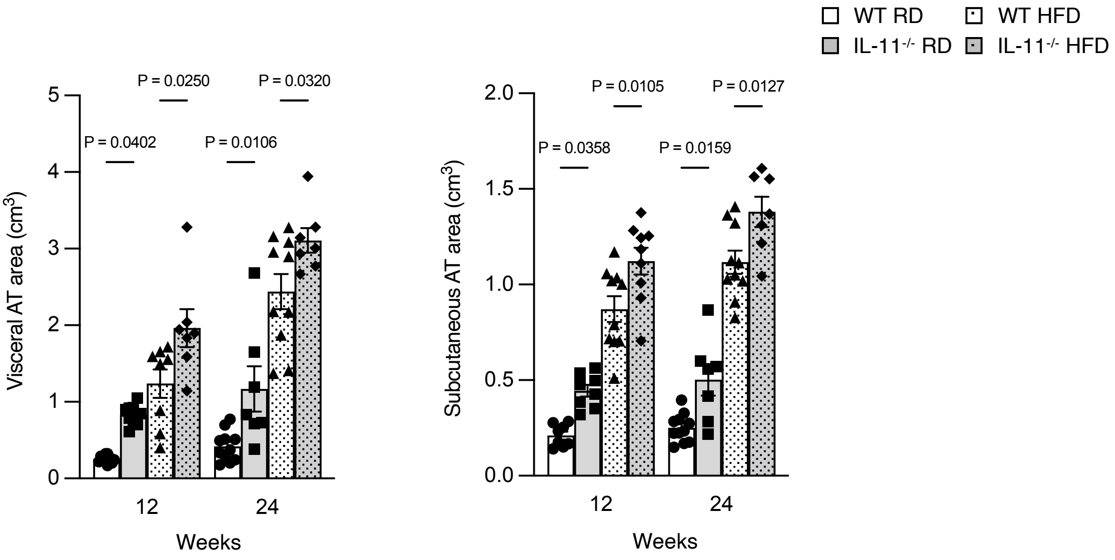

Supplementary Figure 5. Visceral and Subcutaneous adipose tissue area in WT and IL-11$^{-/-}$ mice under RD and HFD at 12 and 24 weeks old.

Fat percentage was measured by micro CT. Visceral AT area at 12 weeks of WT on RD (n = 10) or HFD (n = 8) and of IL-11$^{-/-}$ mice on RD (n = 11) or HFD (n = 7), and at 24 weeks of WT on RD (n = 11) or HFD (n = 10) and of IL-11$^{-/-}$ mice on RD (n = 7) or HFD (n = 7). Subcutaneous AT area at 12 weeks of WT on RD (n = 8) or HFD (n = 10) and of IL-11$^{-/-}$ mice on RD (n = 8) or HFD (n = 9), and at 24 weeks of WT on RD (n = 11) or HFD (n = 10) and of IL-11$^{-/-}$ mice on RD (n = 7) or HFD (n = 7). Data are means $\pm$ SD. P values between WT and IL-11$^{-/-}$ mice under the same diet are calculated by two-way ANOVA with Tukey's multiple comparisons test.

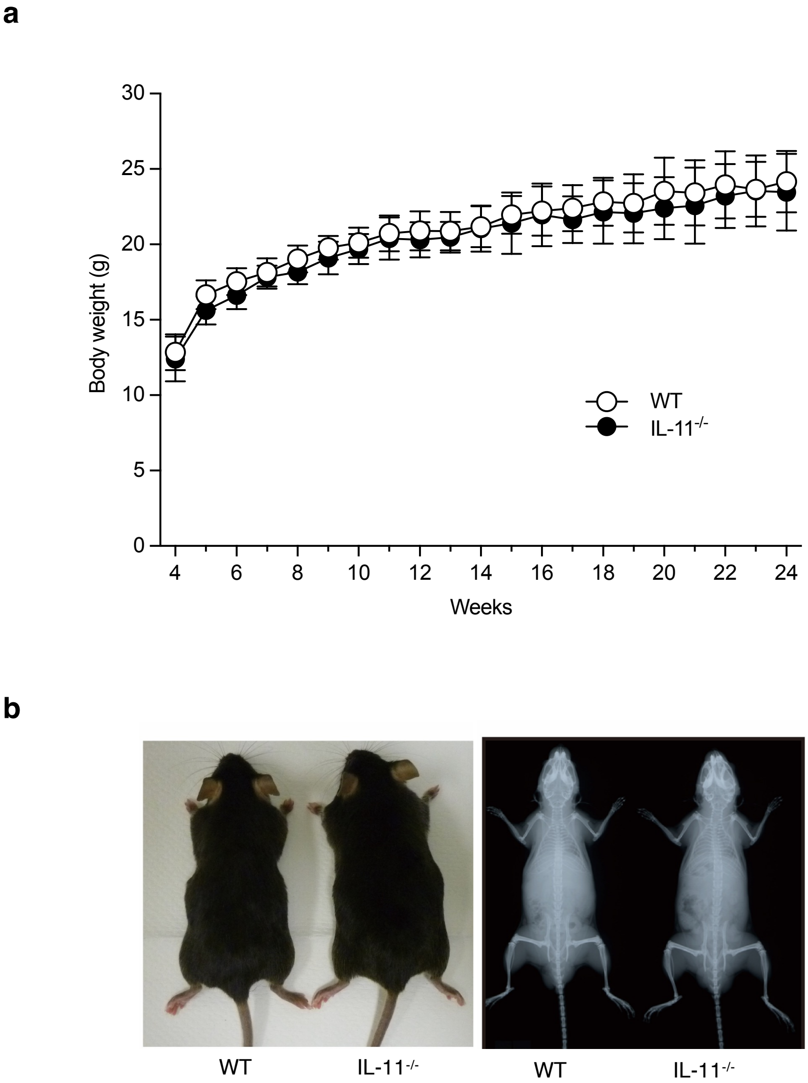

Supplementary Figure 6. Body weight and size of WT and IL-11$^{-/-}$ mice.
(a) Body weight gain in WT (n = 19) and IL-11$^{-/-}$ mice (n = 20). Data are means ± SD. No significant difference was observed between the two groups. **(b)** Body size of WT and IL-11$^{-/-}$ mice at 12 weeks.

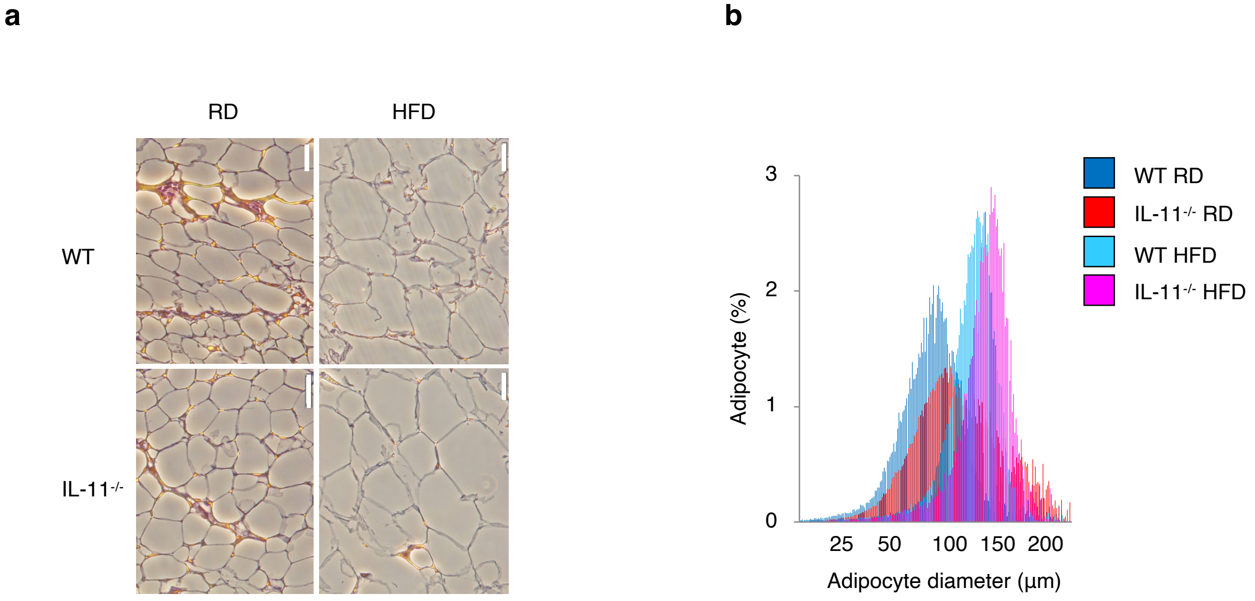

Supplementary Figure 7. Histology of WAT and distribution of adipocyte diameter in WT and IL-11$^{-/-}$ mice on RD and HFD.

(a) H.E. staining of WAT from 32-week-old WT and IL-11$^{-/-}$ mice on either RD or HFD. Representative pictures of three independent experiments. Scale bar = 100 µm. **(b)** Distribution of adipocyte diameter in WT and IL-11$^{-/-}$ mice on RD or HFD.

a

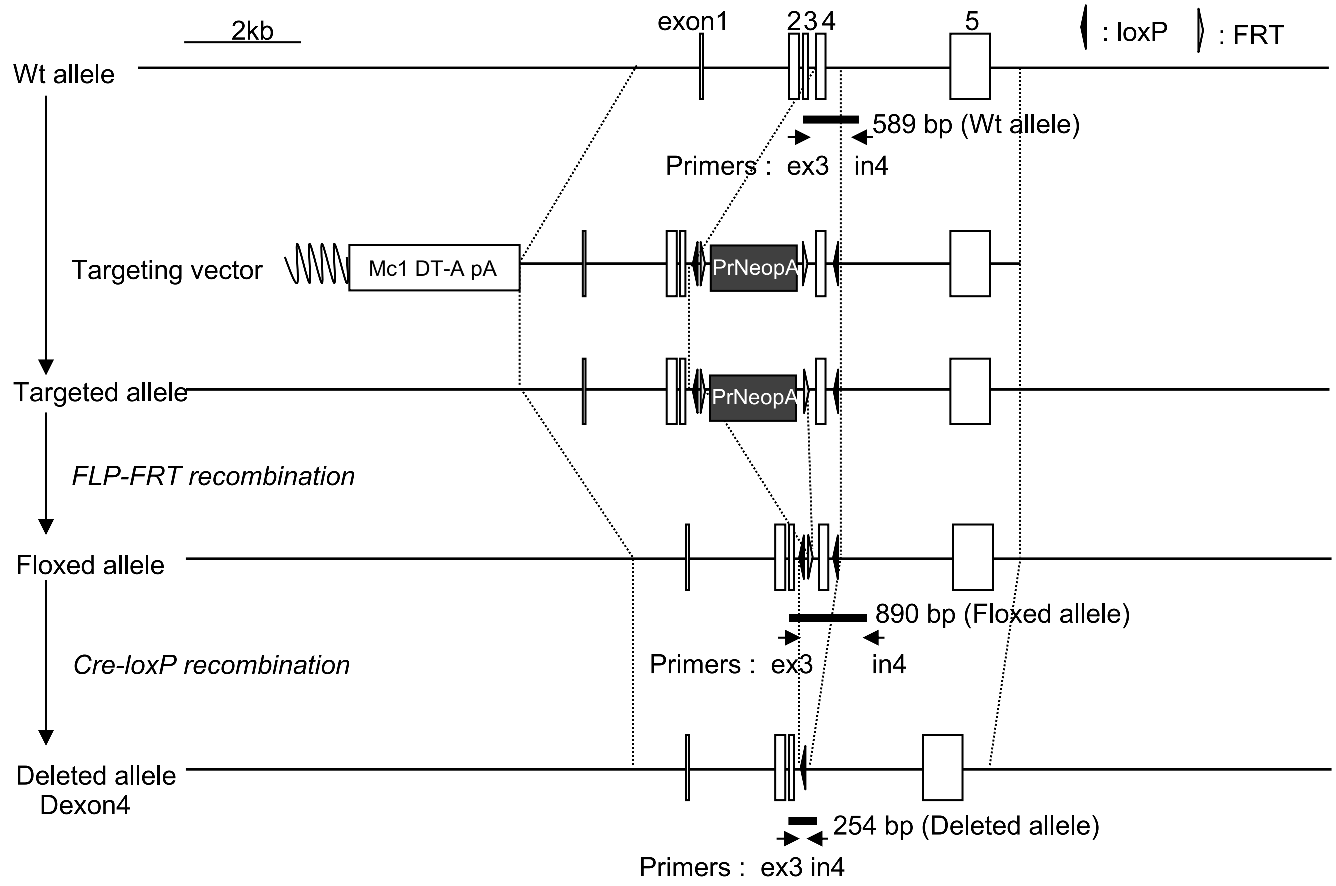

b

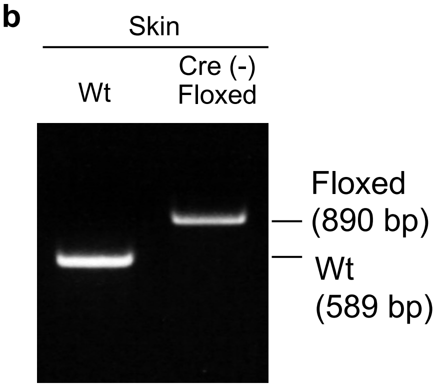

Supplementary Figure 8. Generation of conditional IL-11 knockout mice
(a) Diagrammatic representation of WT allele, targeting vector, targeted allele, floxed allele and deleted allele. Because homozygous IL-11 targeted female mice were infertile, embryo was obtained from these mice and neomycin cassette was deleted using FLP-FRT recombination. (b) PCR analysis of genomic DNA extracted from IL-11$^{w/w}$, IL-11$^{fl/w}$ and IL-11$^{fl/fl}$ mice. A representative picture of three independent experiments.

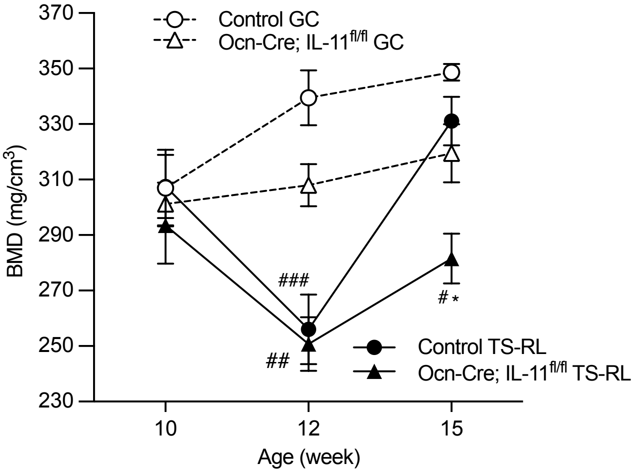

Supplementary Figure 9. Reseponses to mechanical loading and unloading in osteoblast/osteocyte-specific Ocn-Cre;IL-11$^{flox/flod}$ mice are similar to those in systemic IL-11$^{-/-}$ mice.

Vertebral BMD of control and Ocn-Cre;IL-11$^{fl/fl}$ mice in ground control (GC), tail suspension (TS) and reloading (RL) groups. n=6. Data are means ± SE. *P = 0.0021 vs WT, $^{\#}$P = 0.0196, $^{\#\#}$P = 0.0034, $^{\#\#\#}$P < 0.0001 vs GC group in the same genotype using one-way ANOVA with Tukey's multiple comparisons test.

a

b

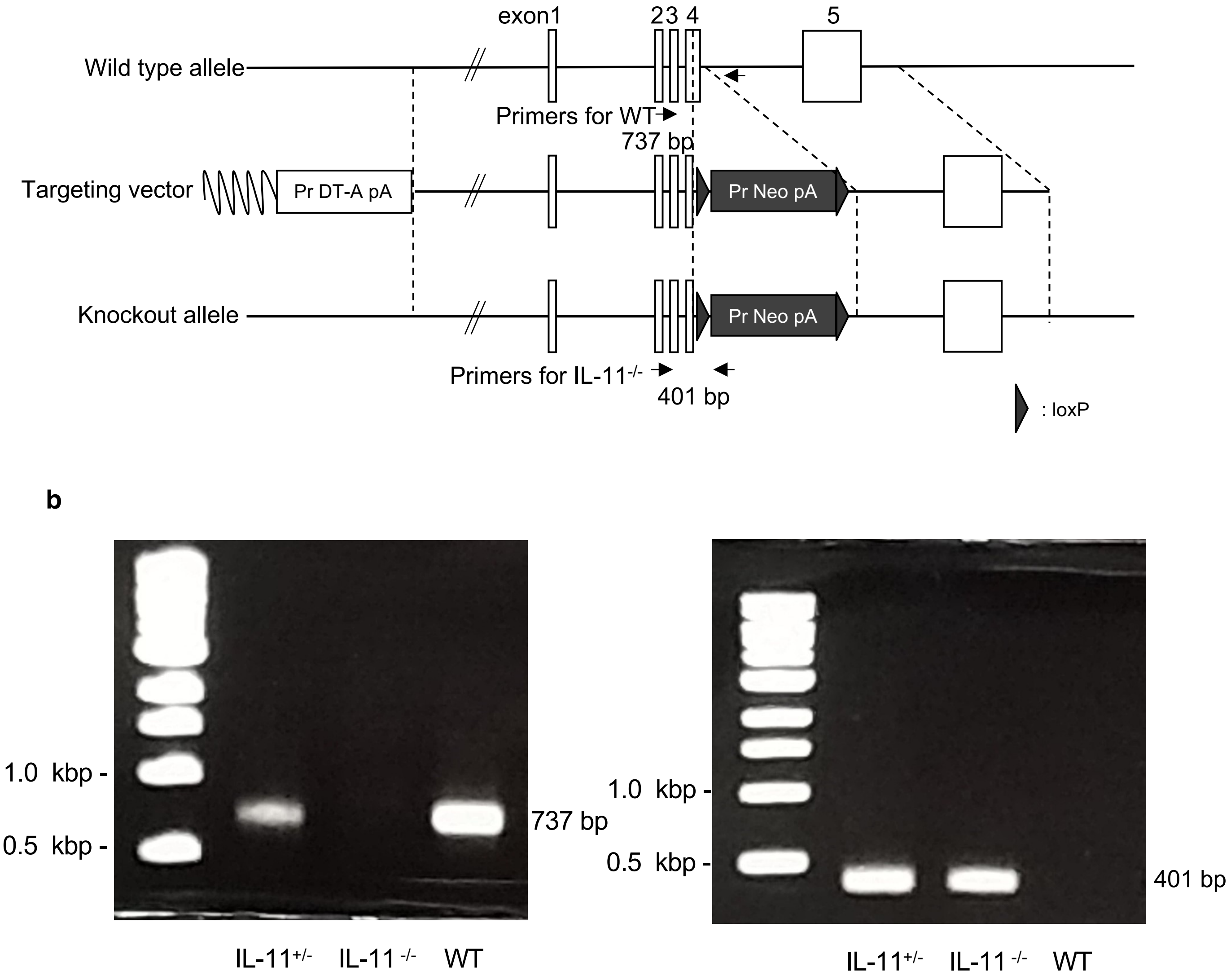

Supplementary Figure 10. Generation of IL-11 knockout mice

(a) Diagrammatic representation of WT allele, targeting vector and mutant allele. (b) PCR analysis of genomic DNA extracted from WT and IL-11$^{-/-}$ mice. Different primer sets were used to detect genomic DNA from WT (left) and IL-11$^{-/-}$ mice (right) as mentioned in the Methods section. Representative pictures of all mice used for experiments.

Supplementary Table 1. Compositions of RD and HFD

	Protein (% calorie)	Fat (% calorie)	Carbohydrate (% calorie)	Total calorie (kcal/100g)
RD	25.7	13.6	60.7	357
HFD	17.2	54.5	28.3	481

Supplementary Table 2. Primer sequences for PCR

Target gene	Forward primer (5'-3')	Reverse primer (5'-3')
Ocn	acagactccggcgctacctt	aatagtgataccgtagatgcgtttg
Runx2	catttgcactgggtcacacgta	gaatctggccatgtttgtgctc
Osx	actcatccctatggctcgtg	ggtagggagctgggttaagg
Rankl	tgcagaaggaactgcaacac	gatggtgaggtgtgcaaatg
Ctsk	cagcagaacggaggcattga	ctttgccgtggcgttatacatac
Trap	cagcagccaaggaggactac	acatagcccacaccgttctc
Sost	cttcaggaatgatgccacagaggt	atctttggcgtcatagggatggtg
Dkk1	cagtgccaccttgaactcagttct	tggtacttgttcccgccctcata
Dkk2	ggaatctgcatcccagtcactgag	tgtggccttcctagattctgccat
Axin2	ctccccaccttgaatgaaga	actgggtcgcttctcttgaa
Ccnd1	gcgtaccctgacaccaatct	ctcttcgcacttctgctcct
Ccnd4	tgatgatgacgcaaggagac	cgggcattgacgttagagat
Pparγ	tgtcggtttcagaagtgccttg	ttcagctggtcgatatcactggag
Cebpα	tggacaagaacagcaacgag	tcactggtcaactccagcac
Hsl	tcaaccgaccaggagtgct	ctcgttgcgtttgtagtgctc
Atgl	cttcctcggggtctaccaca	gcctccttggacacctcaataa
Aco	cagcaggagaaatggatgca	gggcgtaggtgccaattatct
Acc	aacatccccacgctaaacag	ctgacaaggtggcgtgaag
Mcp-1	ccactcacctgctgctactcat	tggtgatcctcttgtagctctcc
Tnfα	ctgtagcccacgtcgtagc	ttgagatccatgccgttg
IL-11	aaattcccagctgacggagatc	tacatgccggaggtaggacatc
IL-11 Rα	acttccacctcaggacatcg	ggtggttaaaaggacaggca
β-actin	tggaatcctgtggcatccatgaaac	taaaacgcagctcagtaacagtccg
Gapdh	aaatggtgaaggtcggtgtg	tgaaggggtcgttgatgg
