# Supplementary material for: Osteoblast/osteocyte-derived interleukin-11 regulates osteogenesis and systemic adipogenesis
Source: Nat Commun. 2022 Nov 23;13:7194. doi: 10.1038/s41467-022-34869-3 (PMC9691688; doi:10.1038/s41467-022-34869-3)
Supplement: Supplementary file 3 — Reporting Summary [file 41467_2022_34869_MOESM3_ESM.pdf]

nature portfolio

Corresponding author(s): Toshio Matsumoto

Last updated by author(s): Oct 20, 2022

Reporting Summary

Nature Portfolio wishes to improve the reproducibility of the work that we publish. This form provides structure for consistency and transparency in reporting. For further information on Nature Portfolio policies, see our Editorial Policies and the Editorial Policy Checklist.

Statistics

For all statistical analyses, confirm that the following items are present in the figure legend, table legend, main text, or Methods section.

n/a	Confirmed	
☐	☒	The exact sample size (n) for each experimental group/condition, given as a discrete number and unit of measurement
☐	☒	A statement on whether measurements were taken from distinct samples or whether the same sample was measured repeatedly
☐	☒	The statistical test(s) used AND whether they are one- or two-sided<br>*Only common tests should be described solely by name; describe more complex techniques in the Methods section.*
☒	☐	A description of all covariates tested
☐	☒	A description of any assumptions or corrections, such as tests of normality and adjustment for multiple comparisons
☐	☒	A full description of the statistical parameters including central tendency (e.g. means) or other basic estimates (e.g. regression coefficient) AND variation (e.g. standard deviation) or associated estimates of uncertainty (e.g. confidence intervals)
☐	☒	For null hypothesis testing, the test statistic (e.g. F, t, r) with confidence intervals, effect sizes, degrees of freedom and P value noted<br>*Give P values as exact values whenever suitable.*
☒	☐	For Bayesian analysis, information on the choice of priors and Markov chain Monte Carlo settings
☒	☐	For hierarchical and complex designs, identification of the appropriate level for tests and full reporting of outcomes
☒	☐	Estimates of effect sizes (e.g. Cohen's d, Pearson's r), indicating how they were calculated

Our web collection on statistics for biologists contains articles on many of the points above.

Software and code

Policy information about availability of computer code

Data collection	For microCT measurements, data were captured and analyzed by Latheta LCT-200 software Version 3.56 (Hitachi-Aloka, Tokyo, Japan).<br>Bone histomorphometry was performed using OsteoMeasure Version 3.2.1.4 (OsteoMetrics, Inc.,GA, USA).<br>Real-time PCR reactions were performed using the ABI 7300-Real-Time PCR System Sequence Detection Software Version 1.4.1 (Applied Biosystems).
Data analysis	Graphpad Prism Version 9.4.1 was used to analyze the data.

For manuscripts utilizing custom algorithms or software that are central to the research but not yet described in published literature, software must be made available to editors and reviewers. We strongly encourage code deposition in a community repository (e.g. GitHub). See the Nature Portfolio guidelines for submitting code & software for further information.

Data

Policy information about availability of data

All manuscripts must include a data availability statement. This statement should provide the following information, where applicable:

- Accession codes, unique identifiers, or web links for publicly available datasets
- A description of any restrictions on data availability
- For clinical datasets or third party data, please ensure that the statement adheres to our policy

Information on the mouse Il11ra locus was acquired from the following web site. (Ensemble ID: ENSMUST00000098132.10, https://www.uniprot.org/uniprotkb/

Q64385/entry)
The data supporting the findings from this study are available within the manuscript and its supplementary information. Source data are provided with this paper.

Human research participants

Policy information about studies involving human research participants and Sex and Gender in Research.

Reporting on sex and gender	N/A
Population characteristics	N/A
Recruitment	N/A
Ethics oversight	N/A

Note that full information on the approval of the study protocol must also be provided in the manuscript.

Field-specific reporting

Please select the one below that is the best fit for your research. If you are not sure, read the appropriate sections before making your selection.

☒ Life sciences ☐ Behavioural & social sciences ☐ Ecological, evolutionary & environmental sciences

For a reference copy of the document with all sections, see nature.com/documents/nr-reporting-summary-flat.pdf

Life sciences study design

All studies must disclose on these points even when the disclosure is negative.

Sample size	No statistical methods were used to calculate sample size. Sample sizes were determined on the basis of previous publications of similar studies (Dudakovic A. et al., 2015, PMID: 26424790; Thaler R. et al., 2016, PMID: 26757819; Khani F. et al., 2017, PMID: 27862226), as well as previous experience with similar type of experiments in our laboratory.
Data exclusions	Animals in poor body condition were excluded.
Replication	In principle, we repeat at least three experiments. If the results of those experiments are inconsistent, we may repeat more experiments. Because all of the data in Figures 2f, 2h, 4a, 6a, 7e and Supple Figures 1c, 7a, and 8b are consistent, we repeated three experiments, and genotyping was performed in all mice used for experiments for Suppl Fig 10b. The information is included in the respective figure legend.
Randomization	Animals were grouped randomly. For in vitro experiments, cells were all plated randomly at the same time, and wells were randomly selected for different treatments. Cell harvesting, processing and analysis was performed in random order.
Blinding	Animals were grouped blindly to researchers. For bone mineral density measurements, technicians were blinded to the sample information. For bone histomorphometric analyses, investigators were unaware of the mouse groups. For immunohistochemistry and western blotting, investigators were blinded to the group allocation during the data collection and blinded to sample identity for the analysis.

Reporting for specific materials, systems and methods

We require information from authors about some types of materials, experimental systems and methods used in many studies. Here, indicate whether each material, system or method listed is relevant to your study. If you are not sure if a list item applies to your research, read the appropriate section before selecting a response.

Materials & experimental systems

n/a	Involved in the study	
☐	☒	Antibodies
☐	☒	Eukaryotic cell lines
☒	☐	Palaeontology and archaeology
☐	☒	Animals and other organisms
☒	☐	Clinical data
☒	☐	Dual use research of concern

Methods

n/a	Involved in the study	
☒	☐	ChIP-seq
☒	☐	Flow cytometry
☒	☐	MRI-based neuroimaging

Antibodies

Antibodies used	The following antibodies were used:<br>Western blot:<br>Phospho-Stat1 (Tyr701) (D4A7) Rabbit mAb (7649, Cell Signaling Technology, 1:1,000),<br>Stat1 (D4Y6Z) Rabbit mAb (14995, Cell Signaling Technology, 1:1,000),<br>Phospho-Stat3 (Tyr705) Antibody (9131, Cell Signaling Technology, 1:1,000),<br>Stat3 (79D7) Rabbit mAb (4904, Cell Signaling Technology, 1:1,000),<br>HDAC4 (D8T3Q) Rabbit mAb (15164, Cell Signaling Technology, 1:1,000),<br>HDAC5 (D1J7V) Rabbit mAb (20458, Cell Signaling Technology, 1:1,000),<br>GAPDH (D16H11) XP® Rabbit mAb (5174, Cell Signaling Technology, 1:1,000),<br>β-Actin (8H10D10) Mouse mAb (3700, Cell Signaling Technology, 1:1,000),<br>Anti-rabbit IgG, HRP-linked Antibody (7074, Cell Signaling Technology, 1:10,000),<br>Anti-mouse IgG, HRP-linked Antibody (7076, Cell Signaling Technology, 1:10,000),<br>Anti-Nuclear Matrix Protein p84 mouse monoclonal antibody [5E10] (ab487, Abcam, 1:1,000)<br><br>Immunohistochemistry:<br>Mouse SOST/Sclerostin Antibody (AF1589, R&D Systems, 1:100)<br>Mouse Dkk-1 Antibody (AF1765SP, R&D Systems, 1:100)<br>Mouse Dkk-2 Antibody (AF2435, R&D Systems, 1:100)
Validation	All antibodies used have been validated by the manufactures. Protocols for western blotting and immunohistochemistry were performed according to the manufacturer's instructions.<br>Western blot:<br>Phospho-Stat1 (Tyr701) (D4A7) Rabbit mAb (7649, Cell Signaling Technology, 1:1000),<br>https://www.cellsignal.jp/products/primary-antibodies/phospho-stat1-tyr701-d4a7-rabbit-mab/7649<br>Stat1 (D4Y6Z) Rabbit mAb (14995, Cell Signaling Technology, 1:1000),<br>https://www.cellsignal.jp/products/primary-antibodies/stat1-d4y6z-rabbit-mab/14995<br>Phospho-Stat3 (Tyr705) Antibody (9131, Cell Signaling Technology, 1:1000),<br>https://www.cellsignal.jp/products/primary-antibodies/phospho-stat3-tyr705-d3a7-xp-rabbit-mab/9145<br>Stat3 (79D7) Rabbit mAb (4904, Cell Signaling Technology, 1:1000),<br>https://www.cellsignal.jp/products/primary-antibodies/stat3-79d7-rabbit-mab/4904<br>HDAC4 (D8T3Q) Rabbit mAb (15164, Cell Signaling Technology, 1:1000),<br>https://www.cellsignal.jp/products/primary-antibodies/hdac4-d8t3q-rabbit-mab/15164<br>HDAC5 (D1J7V) Rabbit mAb (20458, Cell Signaling Technology, 1:1000),<br>https://www.cellsignal.jp/products/primary-antibodies/hdac5-d1j7v-rabbit-mab/20458<br>GAPDH (D16H11) XP® Rabbit mAb (5174, Cell Signaling Technology, 1:1000),<br>https://www.cellsignal.jp/products/primary-antibodies/gapdh-d16h11-xp-rabbit-mab/5174<br>β-Actin (8H10D10) Mouse mAb (3700, Cell Signaling Technology, 1:1000),<br>https://www.cellsignal.jp/products/primary-antibodies/b-actin-8h10d10-mouse-mab/3700<br>Anti-rabbit IgG, HRP-linked Antibody (7074, Cell Signaling Technology, 1:1000),<br>https://en.cellsignal.jp/products/secondary-antibodies/anti-rabbit-igg-hrp-linked-antibody/7074<br>Anti-mouse IgG, HRP-linked Antibody (7076, Cell Signaling Technology, 1:1000),<br>https://en.cellsignal.jp/products/secondary-antibodies/anti-mouse-igg-hrp-linked-antibody/7076<br>Anti-Nuclear Matrix Protein p84 mouse monoclonal antibody [5E10] (ab487, Abcam, 1:1000)<br>https://www.abcam.co.jp/nuclear-matrix-protein-p84-antibody-5e10-ab487.html<br><br>Immunohistochemistry:<br>Mouse SOST/Sclerostin Antibody (AF1589, R&D Systems, 1:100)<br>https://www.rndsystems.com/products/mouse-sost-sclerostin-antibody_af1589<br>Mouse Dkk-1 Antibody (AF1765SP, R&D Systems, 1:100)<br>https://www.rndsystems.com/products/mouse-dkk-1-antibody_af1765<br>Mouse Dkk-2 Antibody (AF2435, R&D Systems, 1:100)<br>https://www.rndsystems.com/products/mouse-dkk-2-antibody_af2435

Eukaryotic cell lines

Policy information about cell lines and Sex and Gender in Research

Cell line source(s)	MC3T3-E1 cell: osteoblastic cells from C57BL/6 mouse calvaria, RIKEN Cell Bank Cat. No. RCB1126<br>C3H10T1/2 cell (clone 8): fibroblastic cells from C3H mouse embryo, RIKEN Cell Bank Cat. No. RCB0247
Authentication	None of the cells used are authenticated
Mycoplasma contamination	All cell lines are tested negative for mycoplasma contamination
Commonly misidentified lines<br>(See ICLAC register)	None

Animals and other research organisms

Policy information about studies involving animals; ARRIVE guidelines recommended for reporting animal research, and Sex and Gender in Research

Laboratory animals	C57BL/6 mouse strain was used in this study. All the mice were housed in SPF conditions, 12 hours light/dark cycle in 22-25°C with 90 ± 5% humidity.<br>TOPGAL transgenic mice that express beta-galactosidase as a reporter in the presence of TCF/LEF-mediated signaling pathway (Strain #004623, RRID: IMSR_JAX:004623) were obtained from the Jackson Laboratory (Bar Harbor, ME, USA).
Wild animals	No wild animals were used.
Reporting on sex	Female mice were used for all the experiments. Total number of mice used in all experiments was not recorded.
Field-collected samples	No field-collected samples were used in this study.
Ethics oversight	All animal experiments were performed according to the guidelines of the Animal Research Committee, the University of Tokushima Graduate School of Health Biosciences and the Institutional Animal Care and Use Committee of RIKEN Kobe Branch.

Note that full information on the approval of the study protocol must also be provided in the manuscript.
